# Deceptive Manipulation of Competitive Starting Strategies Influences Subsequent Pacing, Physiological Status, and Perceptual Responses during Cycling Time Trials

**DOI:** 10.3389/fphys.2016.00536

**Published:** 2016-11-11

**Authors:** Emily L. Williams, Hollie S. Jones, S. Andy Sparks, David C. Marchant, Adrian W. Midgley, Craig A. Bridge, Lars R. McNaughton

**Affiliations:** ^1^School of Sport, Leeds Beckett UniversityLeeds, UK; ^2^School of Psychology, University of Central LancashirePreston, UK; ^3^Sports Performance Group, Edge Hill UniversityOrmskirk, UK

**Keywords:** pacing, time trials, deception, power output, perceived exertion, affect, self-efficacy

## Abstract

Little is currently known regarding competitor influence on pacing at the start of an event and in particular the subsequent effect on the remaining distance. The purpose of the present study was to investigate the influence of starting pace on the physiological and psychological responses during cycling time trials (TT) utilizing an innovative approach allowing pace to be accurately and dynamically replicated, as well as deceptively manipulated. Ten competitive male cyclists completed five 16.1 km TT, two baseline trials performed alone (BLs), and three with a simulated, dynamic avatar of which they were to match the pace of for the initial 4 km. The avatar represented either the cyclist's fastest BL performance (NORM), 105% (FAST), or 95% (SLOW), of fastest BL performance (FBL). Physiological and psychological responses were measured every quartile of the TT. Despite manipulating a starting speed of ± 5% of fastest previous performance, there was no effect on overall 16.1 km TT performance. Manipulated starting strategies did however evoke different physiological and perceptual responses. Whole trial differences found that SLOW produced lower HR, VO_2_, BLa and RPE than FBL (*p* ≤ 0.03) and higher SE than FAST (*p* ≤ 0.03). Additionally, FAST had greater internal attention than NORM (*p* < 0.04). Over time all psychological and physiological variables had a significant condition × quartile interaction in the initial or second quartile mediated by the prescribed starting strategies. Furthermore, RPE, affect, and internal attention remained elevated throughout FAST despite an attenuation in pace during self-selection of pace. There were no differences in performance time when manipulating a 16.1 km cycling TT starting strategy. A slow start, encouraged greater positive perceptions, and less negative physiological consequences than a faster start, and produces no impairment to performance time. It would therefore be considered an advantage in a non-drafting event, not to follow pace of fellow, superior competitors at the start of an event but perform a more negative pacing strategy, with the potential for a greater speed increase against opponents in the latter stages.

## Introduction

Athletes select their starting strategy based on previous experience and task knowledge (Tucker, 2009; Tucker and Noakes, 2009; Smits et al., 2014; Williams et al., 2014b, 2015). Whilst this is the case during solo events, in the initial stages of a competitive race, athletes often do not self-select their pace, but rather adjust their speed to that performed by their opponents (Thiel et al., 2012). Although the athlete may initially envisage an overall pacing schema during an event, schemas are continuously modified in response to external factors such as opponents and tactics (Thiel et al., 2012; Thompson, 2015). Tactics represent dynamic decisions of how and when to invest energy (Smits et al., 2014), together with conscious choices to disrupt their opponent's performance (Thiel et al., 2012). Equally, decisions are made to alter work rate to ensure no harm to physiological status, or to avoid premature termination of the task (Micklewright et al., 2010; Thiel et al., 2012; Williams et al., 2014a). Often this is in response to a poor decision selecting unsustainable starting speeds and clearly supports the importance of interactive psychophysiological decision making (Swart et al., 2009).

Emotional responses play a key role in human decision making (Martino et al., 2006). The reaction to a competitor's movement is based on self-confidence and previous experience in a competitive situation (Foster et al., 1993; Wellner et al., 2010), integrated with the athletes' anxiety, motivation and excitement on the day of the event (St Clair Gibson et al., 2006). In cycling, changes in pacing strategy can significantly affect performance (van Ingen et al., 1992), specifically the exercise intensity elicited during the starting phase of an event (Mattern et al., 2001). It is not, however, well-understood which type of pacing strategy results in the best possible performance, as manipulating starting workloads has been investigated across athletic events of varying durations (Abbiss and Laursen, 2008).

Previous research has employed different distances or durations; <6 min (Aisbett et al., 2009; Bailey et al., 2011), 4–10 km (Gosztyla et al., 2006; Hausswirth et al., 2010; Taylor and Smith, 2014), >10 km (Mattern et al., 2001), and have been assessed within different modes of exercise; running (Gosztyla et al., 2006; Hausswirth et al., 2010), cycling (Mattern et al., 2001; Aisbett et al., 2009; Bailey et al., 2011; Hettinga et al., 2012), or multi-sports such as triathlon (Taylor and Smith, 2014). Furthermore, each have also used diverse magnitudes of increases and decreases in performance intensity (3–15%), and more importantly, they have employed averaged intensity manipulations. Whilst some have used average values from the initial start phases of a self-selected trial (Mattern et al., 2001; Gosztyla et al., 2006), others have included methods with limited ecological validity using whole-trial average manipulations (Aisbett et al., 2009; Hausswirth et al., 2010; Bailey et al., 2011; Hettinga et al., 2012; Taylor and Smith, 2014). The fixed pace nature of the starting strategy will produce conflicting results when compared to trials which are completely self-paced. Equally opponents do not follow a fixed pacing strategy and therefore athletes need to be reactive and responsive to dynamic changes in pace.

Previous research that has used exact, dynamic pacing profiles and investigated different pacing behaviors in both halves of a 4 km TT have found competitor influences (Konings et al., 2016). Observations of athlete behavior has demonstrated the display of intuitive behavior to follow a faster opponent in the opening stages of a race and that a deliberate decision to alter their pacing strategy to compete is evident. The presence of a competitor ostensibly alters the initial 4 km of an athlete's performance in a longer TT (16.1 km TT), whether through motivational, attentional focus (Williams et al., 2014a) or decision making influences (Williams et al., 2015). Previous investigations suggest that a reduced external focus has been observed in the initial 4 km and the final 4 km of a trial where competitors are present (Williams et al., 2014a). Whilst competitors influence attentional focus, it is also well documented that exercise intensity mediates the shift in attentional direction (Hutchinson and Tenenbaum, 2007).

Research is yet to investigate such competitor and intensity influence in a combined setting, specifically in the context of starting strategy manipulations. Knowledge of such effects could help identify the tolerable magnitude of performance increase at the start of an event and the influences on the remaining duration of an event. Furthermore, despite previous research employing starting strategy manipulations and the notion that competitors induce faster starting strategies (Tomazini et al., 2015; Williams et al., 2015), few have examined the perceptual responses of forced starting speeds, and their influence on the subsequent work-rate when able to self-select pace.

The aim of this study was to explore cyclists' responses to an opponent's pace at the start of an event, and specifically investigate the influence of such a starting strategy on the remaining task duration. This would be examined through performance and physiological effects, together with previously unexplored cognitive and perceptual responses. Additionally, the employment of visual avatars to follow as pacers, allowed an exact pacing replication of a previous starting strategy, rather than whole-trial, or starting strategy, average. In accordance with previous, similar literature it was hypothesized that the faster starting strategy would be debilitative to performance (Mattern et al., 2001) and increase negative perceptual responses (Williams et al., 2015).

## Materials and methods

### Participants

Ten competitive male cyclists with the following mean (SD) characteristics, age, 33 (7) year; body mass, 81.9 (6.2) kg; height, 180.1 (5.4) cm; peak power output, 4.8 (0.4) W.kg^−1^; and $\dot{\text{V}}$O_2peak_, 54.0 (3.2) ml·kg^−1^·min^−1^ participated in this study. Participants also had >2 years competitive cycling experience and current training volumes were >9 h per week. The institutional ethics committee approved the study and all participants gave informed consent before completing pre-exercise health screening.

### Experimental design

Participants visited the laboratory on six occasions performing a maximal oxygen uptake procedure and five 16.1 km TT's conducted using a repeated measures, counter balanced design. The trials were performed at the same time of day (±2 h) to minimize circadian variation and were separated with 3–7 days to limit training adaptations. Participants were asked to maintain normal activity and sleep pattern throughout the testing period, and to replicate the same diet for the 24 h preceding each testing session. During the 24 h period prior to each trial, participants refrained from any strenuous exercise, excessive caffeine, or alcohol consumption. In the 2 h before each visit, participants consumed 500 ml of water and refrained from food consumption. Participants were informed that the study was examining the influence of visual feedback during TTs, and were fully debriefed regarding the true nature of the study upon completion of all trials (Jones et al., 2013).

### Peak oxygen uptake

The first visit involved the maximal aerobic capacity test performed on a cycle ergometer (Excalibur Sport Lode, Groningen, Netherlands). Following a 5 min warm-up at 100 W, participants began the protocol at a prescribed resistance in accordance with accepted guidelines (British Cycling, 2003), and 20 W.min^−1^ increments were applied until participants reached volitional exhaustion. Continuous respiratory gas analysis (Oxycon Pro, Jaeger, GmbH Hoechburg, Germany) and heart rate (HR) (Polar Electro OY, Kempele, Finland) were measured throughout. HR_peak_, VO_2peak_ and W_peak_ were calculated as the highest 30s average.

### Time trials

During the following five visits, participants performed a 16.1 km cycling TT on their own bike, mounted on a cycle ergometer (Computrainer Pro, Racermate ONE, Seattle, USA). Participants were informed that different feedback effects were being tested and instructed to complete each TT in the fastest time possible, preparing as if it were a genuine event. No verbal encouragement was given to the participants during any trial in order to prevent inconsistencies in the provision of feedback. Participants were fully debriefed as to the nature of the investigation once all trials had been completed.

The first two TTs were used to establish fastest baseline performance and to familiarize participants with the equipment procedures. Prior to each TT, participants completed a 5 min warm-up at 70% HR_max_, determined from the maximal test, followed by 2 min rest. During the TT the ergometer was interfaced with 3D visual software and calibrated prior to each trial according to manufacturer's instructions. The visual display was projected onto a 230 cm screen positioned 130 cm away from the cyclist's front wheel, with the middle of the screen approximately eye level to the cyclists in a riding position. Whilst performing the initial 4 km during each trial the participants received visual feedback of a road as if they were performing on a flat, road-based 16.1 km TT course and their distance covered in km. Once they had reached 4 km the visual feedback of the road was removed and participants were only able to see their distance covered for the remaining 12 km.

The three final TTs were randomized and counterbalanced in order, with the initial 4 km of each performed with visual avatar, virtual road, current distance covered and distance between rider and avatar each displayed on the screen. Participants were instructed to keep pace with the avatar as closely as possible for the entire first 4 km section, after which the visual display would be removed and they should attempt to complete the remaining 12 km in the fastest time possible. One of the three experimental TTs was performed with an avatar which replicated the exact pacing strategy and speed the participant performed during their previous fastest baseline performance (NORM). A second trial displayed an avatar representing their fastest baseline pacing profile, but at a 5% greater speed (FAST), whilst the third experimental TT displayed an avatar with a speed 5% slower than each participant's fastest baseline pacing profile (SLOW). The manipulation was applied to the speed of the avatar at 34 Hz intervals in order to accurately replicate the exact FBL pacing strategy, ±5% in speed. The participants were not informed as to what the avatar's performance represented, only to follow them as closely as possible. They were reminded to increase their speed to stay with the avatar during the trial if the gap between themselves and the avatar was >10 m.

### Experimental measures

Power output, speed and elapsed time were obscured from the view of the participants throughout the TT, stored and each subsequently downloaded for analysis. Heart rate was also blinded and recorded continuously using the Polar team system sampled at 5 s frequencies and averaged as quartile data points for analysis. During each TT, breath-by-breath respiratory gasses were measured for the duration of a kilometer at every 4 km (e.g., 3.5–4.5 km), expressed in 5 s intervals and subsequently averaged for each quartile analysis. Finger-tip blood lactate (BLa) was also collected at the end of each 4 km quartile during the time trials. Participants were asked to remain in their usual cycling position whilst a capillary blood sample was procured from the finger-tip during the trial (Lactate pro Two LT-1730, Arkray, Japan).

During the initial 4 km participants were asked to rate their perceived exertion (RPE) on a 6–20 scale Borg scale (Borg, 1970), and their affect every kilometer. Affective feeling states (Hardy and Rejeski, 1989) indicating whether exercise felt pleasant or unpleasant, was measured using an 11-point Likert scale ranging from −5 to +5 with verbal anchors at all odd integers and zero (+5 = very good, +3 = good, +1 = fairly good, 0 = neutral, −1 = fairly bad, −3 = bad, –5 = very bad). For the following 12 km participants were asked to rate their RPE, affect, self-efficacy and attentional focus every 4 km. Participant's self-efficacy to continue at the current pace (SE_pace_) was recorded on a 0–100% scale divided into 5% integer intervals. The scale was adopted as previously recommended (Bandura, 1977), with the questions constructed specific to the task due to perform. Attentional focus was recorded using a 10-point Likert scale (Tenenbaum and Connolly, 2008), with participants asked to indicate where their attention had been focused over the last kilometer in relation to external and internal thoughts. Attentional focus was also measured retrospectively, as a maintenance check, once the trial was completed. This was recorded as a percentage of attention that was focused on internal thoughts during different distances (whole-trial, 0–4, 4–8, 8–12, and 12–16.1 km).

### Statistical analysis

Normality was assessed using Shapiro-Wilk approach suitable for the sample size used. Paired *t*-tests were performed to analyse the presence of any systematic bias between the two baseline trials. Only the faster of the two baselines (FBL) was included in the inferential analysis. Six participants performed their fastest baseline in their first baseline trial and the four in their second baseline suggesting that any learning effect was not sufficient to significantly influence overall performance time. The effects of condition (FBL, NORM, FAST, SLOW) and distance quartile (0–4, 4–8, 8–12, and 12–16.1 km) on completion time, PO, speed, HR, RPE, affect, self-efficacy, attentional focus, blood lactate and VO_2_ were analyzed using the Mixed procedure for repeated measures (Peugh, 2005). Various plausible covariance structures were assumed for each dependent variable and the one that minimised the Hurvich and Tsai's criterion (AICC) value was chosen as the best fitting and used for the final model. A quadratic term for distance quartile was entered into the model where appropriate and removed where no significance value was observed. *Post hoc* pairwise comparisons with Sidak-adjusted *p*-values were conducted where a significant F ratio was observed. Statistical significance was accepted as *p* < 0.05 (IBM Statistics 22.0; SPSS Inc., Chicago, IL).

## Results

Across all conditions there was no significant main effect for condition (*F* = 0.8, *p* = 0.51) observed for TT time (Table 1). There were no significant differences in time (*t*_9_ = 0.53; *p* = 0.6), speed (*t*_9_ = −0.35, *p* = 0.7), power output (*t*_9_ = −1.18, *p* = 0.3), heart rate (*t*_9_ = 1.08, *p* = 0.3), RPE (*t*_9_ = 0.0, *p* = 0.1), affect (*t*_9_ = 0.32, *p* = 0.08), self-efficacy (*t*_9_ = 1.18, *p* = 0.3), or attention (*t*_9_ = −0.42, *p* ≥ 0.07) between the two familiarization TT.

**Table 1:** **Mean ± SD values for whole trial variables during each trial condition**.

	**FBL**	**NORM**	**FAST**	**SLOW**
Time (mins)	26.6±1.0	26.8±1.2	26.5±0.9	26.7±1.1
Speed (km.h^−1^)	36.4±1.4	36.0±1.5	36.5±1.2	36.2±1.5
Power output (W)	259±26	252±28	260±15	255±26
Heart Rate (bpm)	161±14	155±14	159±15	154±16

### Starting strategy

There was a main effect for condition for initial 4 km time (*F* = 769.5, *p* < 0.001) with all conditions significantly faster than SLOW (*p* < 0.001) and all conditions significantly slower than FAST (*p* < 0.001). There was no significant difference between FBL and NORM (MD = −0.007, CL = −0.1, 0.9; *p* = 1.0) (Table 2). During the initial 4 km of the FAST and SLOW TT, participants actually rode at 3.6 ± 1.9% above and 5.0 ± 0.1% below fastest baseline speed, respectively. Two participants were unable to keep the <10 m gap during 2–4 km in the FAST trial.

**Table 2:** **Mean ± SD values for the initial quartile during each starting strategy conditions**.

	**FBL**	**NORM**	**FAST**	**SLOW**
Time (mins)	6.6±0.3^*^^#^	6.6±0.3^*^^#^	6.4±0.2^*^	6.9±0.3^#^
Power output (W)	264±29^*^	263±29^*^	290±28^*^	231±25
Speed (km.h^−1^)	36.4±1.4^*^	36.3±1.4	37.7±1.3^*^	34.6±1.4
Bla (mmol.l^−1^)	7.3±2.7^*^	6.4±2.4^*^^#^	9.2±3.2^*^	3.5±1.1
Heart rate (bpm)	153±12	150±14	153±13	140±16
RER	1.15±0.05	1.16±0.06	1.19±0.04	1.15±0.04
$\dot{\text{V}}$E (ml.min^−1^)	120.9±27.9^*^^#^	123.4±26.4^*^^#^	147.1±28.8^*^	99.6±17.8^#^
$\dot{\text{V}}$O_2_ (ml·kg^−1^·min^−1^)	44.2±5.0^*^	43.7±3.9^*^	45.9±9.3	38.8±4.1
Affect	0.45±2.2	0.19±1.8	−0.9±1.7^*^	0.95±1.6
Attention (%)	65.2±31.2	27.5±21.5	69.2±28.1^*^	27.5±23.7
RPE	16.6±1.5^*^	16.0±1.9	16.9±1.8^*^	15.0±1.8
SE (%)	82.5±23.6^#^	85.5±24.8^#^	57.5±35.7	100.0±0.0^#^

* Denotes significantly different to SLOW (p < 0.05);

# *denotes significantly (p < 0.05) different to FAST*.

### Whole-trial

**Figure 1:**
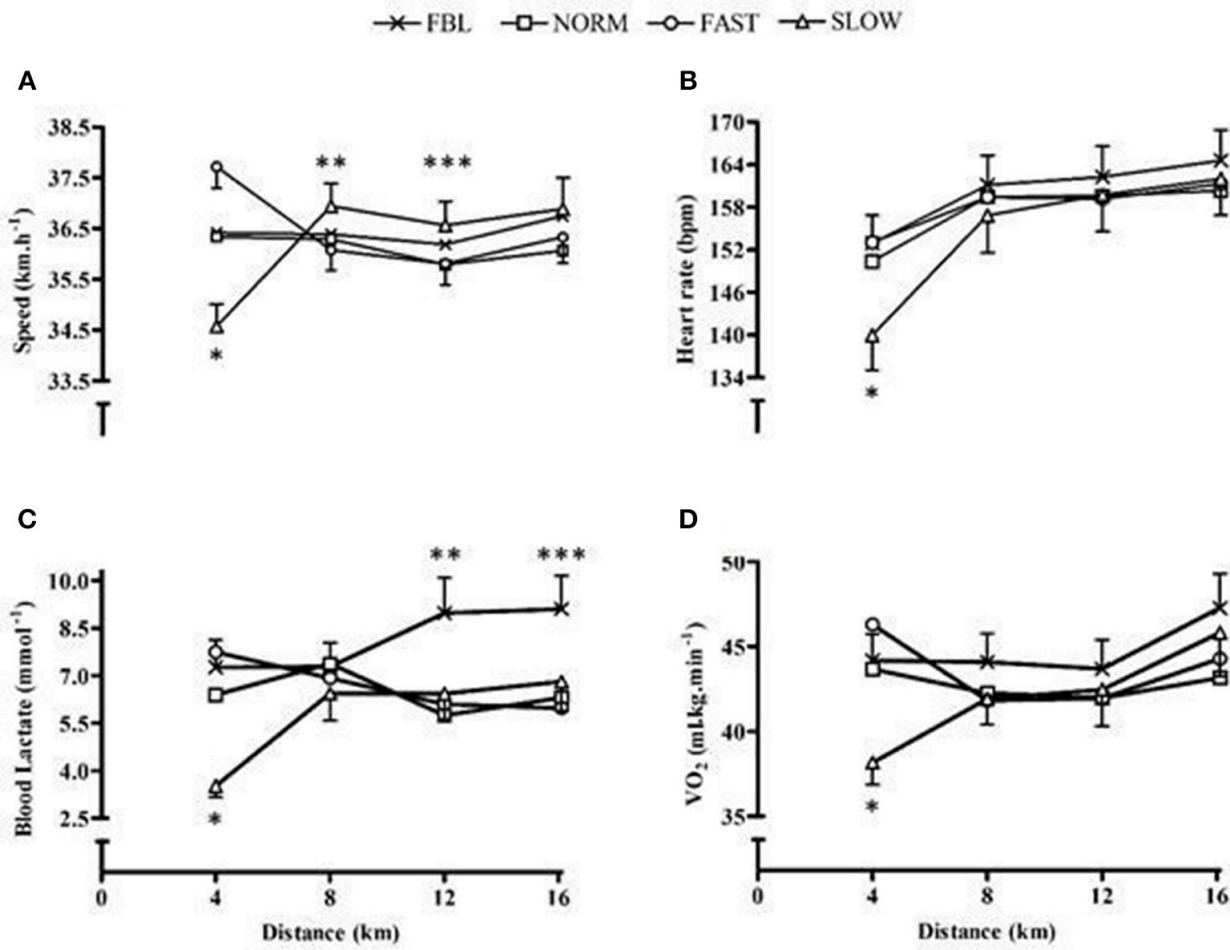
**Whole-trial mean and SEM physiological responses for each condition across distance quartiles, illustrating significant interaction effects**. **(A)** Speed ^*^denotes SLOW significantly slower than both FBL and FAST (*p* ≤ 0.018), ^**^denotes SLOW significantly faster than FAST (*p* = 0.028), ^***^denotes SLOW significantly faster than both NORM and FAST (*p* ≤ 0.01); **(B)** Heart rate, ^*^denotes significantly lower heart rate in SLOW than all other conditions (*p* ≤ 0.001); **(C)** Blood lactate, ^*^ denotes significantly lower values during SLOW than all other conditions (*p* ≤ 0.02), ^**^ denotes significantly higher values in FBL than all other conditions (*p* ≤ 0.04), ^***^denotes significantly higher values in FBL than NORM (*p* = 0.02); **(D)** $\dot{\text{V}}$O_2_, ^*^denotes significantly lower $\dot{\text{V}}$O_2_ during SLOW than FBL and NORM (*p* ≤ 0.02).

Speed had a significant main effect for quartile (*F* = 8.5, *p* = 0.006) and a significant condition × quartile interaction (*F* = 7.8, *p* < 0.001), but no main effect for condition (*F* = 1.5, *p* = 0.26). The third quartile was significantly slower in speed than the second and fourth (*p* ≤ 0.005). *Post hoc* analysis of the interaction effect illustrated that during the first quartile SLOW speed was significantly slower than FBL and FAST (*p* ≤ 0.02). During the second quartile and third quartile SLOW was performed with a significantly faster speed than FAST (*p* = 0.03), and during the last quartile SLOW was performed at a significantly faster speed than FAST and NORM (*p* ≤ 0.01) (Figure 1A).

Power output had a significant main effect for quartile (*F* = 6.8, *p* < 0.001) and a significant condition × quartile interaction (*F* = 14.7, *p* < 0.001), however there was no main effect for condition (*F* = 1.8, *p* = 0.2). The third quartile was performed with significantly less power than the first and fourth (*p* ≤ 0.002). *Post hoc* analysis of the interaction found that FAST, FBL and NORM, during the first quartile, had significantly greater power than SLOW (*p* < 0.001), but during the second quartile there was a significantly greater power performed during SLOW than FAST (*p* < 0.02).

### Physiological responses

There was a significant main effect for condition (*F* = 5.2, *p* = 0.009), quartile (*F* = 41.9, *p* < 0.001) and condition × quartile interaction (*F* = 12.4, *p* < 0.001) for HR. SLOW had a significantly lower HR than FBL (MD = 5.4, CL = 0.4, 10.8; *p* = 0.03) and FAST (MD = 3.6, CL = 0.7, 6.5; *p* = 0.01). There was a significantly lower HR in the first quartile than the remainder of the TT (*p* < 0.001). The interaction *post hoc* analysis illustrated during the first quartile SLOW was performed with a significantly lower HR than all other conditions (*p* < 0.001) (Figure 1B).

There was a significant difference in blood lactate between trials (*F* = 10.8, *p* < 0.001), with lower values produced during SLOW than FBL, NORM, FAST (*p* ≤ 0.002). There was no significant main effect for quartile (*F* = 1.2, *p* = 0.33), however there was a significant condition × quartile interaction (*F* = 3.8, *p* < 0.001) (Figure 1C). A significant main effect for condition (*F* = 0.01, *p* = 0.01) and quartile (*F* = 10.7, *p* < 0.001) and a significant interaction (*F* = 9.0, *p* < 0.001) was identified for VE. FAST had a significantly greater VE than SLOW (MD = 12.9, CL = 2.8, 23.1; *p* = 0.007) and VE significantly increased over time (*p* ≤ 0.002). The *post hoc* analysis for the interaction illustrated during the initial quartile FAST was significantly higher and SLOW was significantly lower than all NORM and FBL (*p* ≤ 0.001). There was no significant main effect for condition for VO_2_ (*F* = 2.9, p = 0.06), but a main effect for quartile (*F* = 7.6, *p* = 0.001) and a significant interaction effect (*F* = 3.3, *p* = 0.008) (Figure 1D). There was also a significant random intercept (*p* = 0.04). *Post hoc* analysis illustrated $\dot{\text{V}}$O_2_ significantly increase over time (*p* ≤ 0.03) and during the initial quartile of SLOW participant's $\dot{\text{V}}$O_2_ was significantly lower than FBL (*p* = 0.01) and NORM (*p* = 0.02). RER values did not have a significant main effect for condition (*F* = 1.2, *p* = 0.31), however a significant main effect for quartile (*F* = 8.2, *p* = 0.001), a significant interaction effect (*F* = 3.9, *p* = 0.004) and a significant random slope (*p* = 0.03) identifying participants having different RER patterns. Pairwise comparisons of the interaction effect showed that during the second quartile FAST had a significantly lower RER than SLOW.

### Psychological responses

**Figure 2:**
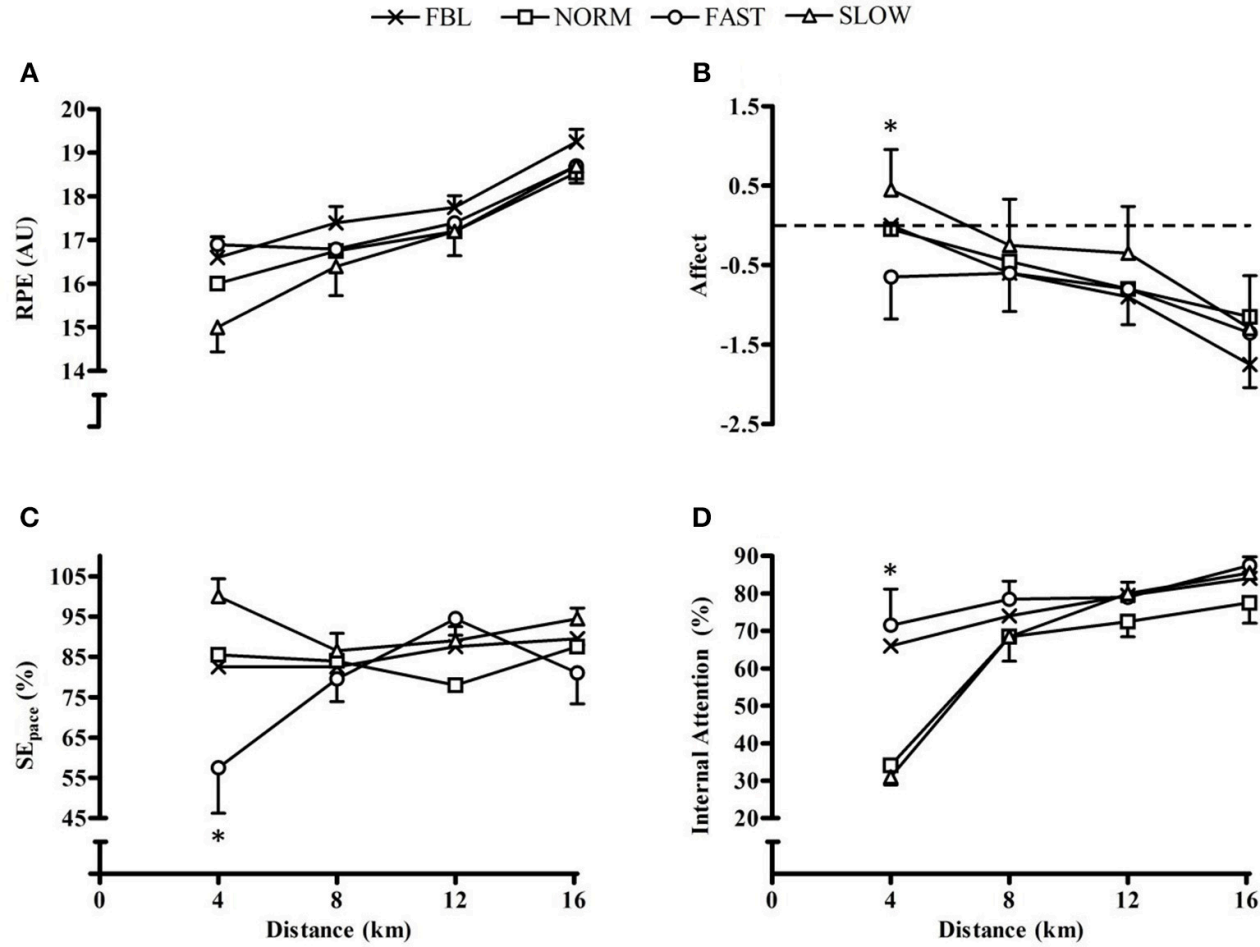
**Whole-trial mean and SEM psychological responses for each condition across distance quartiles, illustrating significant interaction effects**. **(A)** RPE; **(B)** Affect, ^*^denotes significantly lower affect in SLOW than both FBL and FAST (*p* ≤ 0.002); **(C)** SE_pace_, ^*^denotes significantly lower SE_pace_ during FAST than all other conditions (*p* ≤ 0.001); and **(D)** Attentional focus, ^*^denotes significantly higher internal attention during FBL than NORM (*p* = 0.003).

RPE had a significant main effect for condition (*F* = 8.1, *p* = 0.001), quartile (*F* = 37.5, *p* < 0.001) and interaction effect (*F* = 2.5, *p* = 0.02) (Figure 2A). There was a significantly greater RPE during FBL than NORM (MD = 0.6, CL = 0.04, 1.2; *p* = 0.03) and SLOW (MD = 0.9, CL = 0.08, 1.8; *p* = 0.03). During the initial quartile RPE was significantly lower in SLOW than FBL and FAST (*p* ≤ 0.002). Affect was observed to have a significant main effect for quartile (*F* = 11.8, *p* < 0.001), with the final quartile having a significantly lower affect compared to the first and second quartile (*p* ≤ 0.005) (Figure 2B). However, there was no main effect for condition (*F* = 1.5, *p* = 0.24) or an interaction effect (*F* = 1.6, *p* = 0.17).

Self-efficacy had a significant main effect for condition (*F* = 10.7, *p* < 0.001) and a significant condition × quartile interaction (*F* = 3.5, *p* = 0.002), but no main effect for quartile (*F* = 1.4, *p* = 0.27). *Post hoc* analysis found significantly lower SE during FAST than SLOW (MD = −16.9, CL = −25.9, −7.8; *p* < 0.001), and during the first quartile FAST has significantly lower SE than all conditions (*p* ≤ 0.001) (Figure 2C). There was a main effect for condition for during-trial attentional focus (*F* = 5.2, *p* = 0.005). FAST had significantly greater internal attentional focus than NORM (MD = 16.0, CL = 1.0, 30.9; *p* = 0.03). There was a significant main effect for quartile (*F* = 24.2, *p* < 0.001) with the first quartile having significantly lower internal attention than the other three (*p* < 0.001) and the fourth having significantly greater internal attention than all other quartiles (*p* ≤ 0.04). There was also a significant interaction (*F* = 2.1, *p* = 0.05) as during the initial 4 km there was significantly greater internal attentional focus during FBL than NORM (MD = 31.8, CL = 9.6, 54.0; *p* = 0.003) (Figure 2D).

Post-trial attentional focus had a significant main effects for condition (*F* = 4.2, *p* = 0.02), quartile (*F* = 18.3, *p* < 0.001) and an interaction effect (*F* = 7.7, *p* < 0.001). There was significantly greater internal attentional focus during FAST than NORM (MD = 12.6, CL = 0.3, 25.1; *p* = 0.04). The first 4 km had significantly less internal attention than all other time points (*p* < 0.001). In the first 4 km FBL had greater internal attention than NORM (MD = 37.7, CL = 17.5, 57.9; *p* < 0.001) and SLOW (MD = 37.7, CL = 16.8, 58.6; *p* < 0.001). FAST had greater internal attention than NORM (MD = 41.7, CL = 21.5, 61.9; *p* < 0.001) and SLOW (MD = 41.7, CL = 21.5, 6.9; *p* < 0.001).

## Discussion

This study was the first to represent the influence of following a dynamic pace enforced by a fellow cyclist during the opening stages of a 16.1 km TT, and its influence on subsequent pace and previously unexplored perceptual responses. Enforcing manipulated starting speeds of ± 5% of FBL did not affect overall 16.1 km TT performance. Although performances were not significantly altered, pacing strategy decisions, physiological and psychological responses were different and dependent on the starting intensity. As prescribed by the avatar's pace in the initial 4 km performance, data confirm that different starting strategies were effectively enforced during manipulated TTs with significantly slower speeds performed in the initial 4 km during SLOW compared to FBL and FAST. Performing the accurate BL starting strategy (NORM), in comparison to FBL, resulted in no differences in performance or physiological responses, and elicited comparable RPE and affect, however, different psychological responses were observed (self-efficacy and attentional focus). Performing a slower start was associated with a lower RPE than all other starting strategies and a more negative affect compared to the faster start. Such response was observed, not only in the initial 4 km, but also for the whole trial, reflecting the relationship between RPE and intensity (St Clair Gibson et al., 2006). This importantly illustrates that the effects of starting strategy intensity on the RPE and perceptions during the remainder of a TT of this distance.

Although changes in starting strategies produced no differences in overall completion time, speed or power between trials, the subsequent pacing profiles for each trial differed depending on the relative speed maintained during the initial 4 km. During SLOW, participants produced greater speed during the remaining 12 km than all other starting condition trials. In contrast, during FAST, participants decreased their speed during the second quartile, significantly slower than the SLOW trial. These results demonstrated that cyclists made a decision, in both conditions, to change their pace after a forced starting strategy (Renfree et al., 2014; Smits et al., 2014). Cyclists were unable to maintain the elevated speed required during FAST and therefore had to reduce the intensity during the remaining 12 km by 4.5%, in comparison to an increase in the remaining 12 km during SLOW; 2.1% and NORM trials; 2.3%. This emphasizes differences in exertional decision making during competition if athletes are to deviate away from typical, optimal pacing strategies, particularly at the start.

During FAST, the initially elevated physiological responses (HR, VE, and BLa) and RPE, were attenuated after the starting 4 km, but still produced significantly higher values overall compared to SLOW. Previous research has suggested that such responses in the initial quartile could have had a prolonged effect on the remaining duration, with participants unable to recover adequately during the trial (Mattern et al., 2001; Hettinga et al., 2012). This could explain the present study's fast start not facilitating overall performance, despite decreasing performance time during the initial 4 km. Additionally, the observed significantly greater internal attentional focus could have been induced through conscious attempts to regulate effort. Moreover, an increase in starting speed producing lower self-efficacy, could have been due to uncertainty, either linked with limited prior experience of such a starting pace, or concern over elevated physiological feedback and its potential negative effect. This suggests that whilst pace and performance declined when participants were able to self-select workload, the subsequent cognitive, perceptual and physiological responses were arbitrated by the responses to the initial enforced pace (Mattern et al., 2001). The present results also suggest that a 5% increase in self-selected intensity at the start of a 16.1 km TT is too great for the level of rider used in this particular study to sustain. This pace manipulation was unable to be performed with the average increase in pace in FAST at 3.6%. This is important to note for future deception manipulations and particularly stresses the difference in this manipulation to those using fixed power or speed. Whilst using fixed paced manipulation would avoid the variation in manipulation observed in the current study. Using fixed, less ecologically valid methods may be more exposed to athletes detecting the deception and perhaps would lead to greater negative changes in pace, greater physiological disturbance, and worse psychological feeling states in the subsequent duration of the event. Equally leaving the choice to the participant as to whether they would follow the pace of the lead opponent would also increase variations in starting intensities and limit the overall effects found on subsequent pace.

The present study enhances knowledge regarding the influences of performing an initial pace enforced by competition and the interesting psychological processes associated with different starting stratgies; but they dispute previous proposals of a debilitative conservative starting pace (Aisbett et al., 2009; Lima-Silva et al., 2010; Bailey et al., 2011; Hettinga et al., 2012). It has been thought that a conservative starting pace increases the risk of not producing an optimal completion time (Smits et al., 2014), and it has also been suggested that it can be a high-risk strategy to not follow superior competitors at the start of a race (Renfree et al., 2014). The present results demonstrate, that there is no detriment to completion time during a 16.1 km TT if a 5% slower initial speed is adopted in the initial starting phase. This study shows that an initial slower start decreases the initial accumulation of metabolites and the heightened physiological responses allowing workload to be increased during the remaining self-paced 12 km. This decision to increase pace, possibly due to lower physiological strain (HR, $\dot{\text{V}}$O_2_, VE, BLa), and the more positive psychological responses (reduced RPE and internal attention, and improved affect and self-efficacy), lessens the effect of a slower start. This not only enables a similar performance time, but participants also continued to have more positive cognitive and perceptual responses during the remainder of the trial. Such findings promote the advantages of a slow start in non-drafted TTs and highlight important associations with athlete motivation, ability and likelihood of producing a greater endspurt in an event, perhaps when opponents are experiencing more fatiguing symptoms. It would be of interest to investigate such manipulation effects during shorter or longer duration events where there is less or more distance to catch-up after a slower start. Similarly, they offer training and future performance implications suggestive of athlete's adherence to high intensities whether through enjoyment or motivation if the initial workload is lowered. Of interest would be the investigation into this effect during an event which provides efficiencies from drafting a competitor, whereby a similar manipulation could explore at what intensity would the efficiencies gained from drafting not warrant the extra energy to keep with your opponent.

A further aim of this work was to expand on previous research investigating the presence of a competitors influencing attentional focus during exertion (Williams et al., 2014a). Reductions in internal attentional focus were previously shown to inhibit the rise in perceived exertion during performance in the presence of competitors. Differences seen in RPE and internal attentional focus in the initial 4 km between FBL and NORM using the same pacing profile, in the present study, support the previous investigation. Additionally, exercise intensity has been proposed as a mediator of attentional focus (Hutchinson and Tenenbaum, 2007), and this was also observed in the present results, since the presence of a faster avatar and the prescribed increase in intensity, was insufficient to draw attention externally and failed to prevent a rise in perceived exertion. Furthermore, in the presence of a slower avatar, internal attentional focus and RPE were significantly reduced compared to no avatar, or a faster avatar. This could however be because the riders were not asked to compete with the avatar, but to simply match its pace. The importance of instructions may explain differential results to previous research since, the impact of a competitive environment is likely to have additional influences (Schunk, 1995), other than a visual display that would encourage an external focus. Motivational processes have been previously explored in specific events that serve to direct attentional focus toward sources of information, from which it was observed that the motivational influence on attention mechanisms, adaptively regulates perceptual and conceptual processes (van der Linden and Eling, 2006; Williams et al., 2014b). This has been previously found in an experiment with athletes perceiving a great performance when deceived to be performing in the presence of a non-competitive, experimental accomplice (Bath et al., 2012). In addition to the present investigation, motivation could be further explored as to whether its influence changes at different stages of a competition or task duration (i.e., start or end of the trial) or within competitive and non-competitive scenarios.

## Conclusion

These results suggest that with no detriment to performance time, but less physiological strain and more positive psychological perceptions, a pacing strategy adopting a slower start could be considered more beneficial during a stimulated 16.1 km cycling TT. Despite beginning the TT with a conservative pace, resulting in a performance disadvantage of ~18 s, participants were able to overcome this deficit, when they self-selected their subsequent pace. Not only were they able to produce a similar completion time, but also had more positive perceptual responses; reduced RPE and greater affect. Similarly, whilst the view is that attention and affect are dynamic in the face of task progression, the result suggest during high-effort tasks, non-preferable changes in such state may be difficult to recover. This perhaps indicates the necessity for directed cognitive interventions within-task to aid the reversal of the detrimental psychological responses accompanying a fast start.

Practically, these findings highlight the possible importance of not following a pacing strategy that is influenced by lead competitors when unable to gain efficiencies through drafting, since the detrimental impact could be prevented and still enable an equivalent performance. The ability to dictate an early pace on fellow competitors may be beneficial through the impact on cognitive and perceptual responses and their associated influence on pacing decisions. Importantly, a chosen starting strategy has residual influences on the remaining distance, with both physiological responses and perceptual valence determined by the prescribed starting strategies.

## Author contributions

Each author (EW, HJ, SS, DM, CB, AM, LM) contributed to the conception or design of the work. EW, HJ and AM contributed to the analysis and EW, HJ, AM, SS, DM, CB, LM the interpretation of data for the work. Each author (EW, HJ, SS, DM, CB, AM, LM) contributed to the drafting the work and revising it critically for important intellectual content. Each author (EW, HJ, SS, DM, CB, AM, LM) contributed to the final approval of the version to be published; and Each author (EW, HJ, SS, DM, CB, AM, LM) is in agreement to be accountable for all aspects of the work.

### Conflict of interest statement

The authors declare that the research was conducted in the absence of any commercial or financial relationships that could be construed as a potential conflict of interest.
